# Inducible Costimulator Expression Regulates the Magnitude of Th2-Mediated Airway Inflammation by Regulating the Number of Th2 Cells

**DOI:** 10.1371/journal.pone.0007525

**Published:** 2009-11-04

**Authors:** Bryan S. Clay, Rebecca A. Shilling, Hozefa S. Bandukwala, Tamson V. Moore, Judy L. Cannon, Andrew A. Welcher, Joel V. Weinstock, Anne I. Sperling

**Affiliations:** 1 Committee on Immunology, University of Chicago, Chicago, Illinois, United States of America; 2 Section of Pulmonary and Critical Care Medicine, Department of Medicine, University of Chicago, Chicago, Illinois, United States of America; 3 Amgen Inc., Thousand Oaks, California, United States of America; 4 Division of Gastroenterology, Department of Internal Medicine, Tufts New England Medical Center, Boston, Massachusetts, United States of America; New York University School of Medicine, United States of America

## Abstract

**Background:**

Inducible Costimulator (ICOS) is an important regulator of Th2 lymphocyte function and a potential immunotherapeutic target for allergy and asthma. A SNP in the *ICOS* 5′ promoter in humans is associated with increased atopy and serum IgE in a founder population and increased ICOS surface expression and Th2 cytokine production from peripheral blood mononuclear cells. However, it is unknown if increased ICOS expression contributes to disease progression or is a result of disease pathology.

**Methodology/Principal Findings:**

We developed a mouse model in which ICOS surface expression levels are genetically predetermined to test our hypothesis that genetic regulation of ICOS expression controls the severity of Th2 responses *in vivo*. Using ICOS^+/+^ and ICOS^+/−^ mice in a Th2 model of airway inflammation, we found that T cells from the ICOS^+/−^ mice had reduced ICOS expression and decreased Th2-mediated inflammation *in vivo*. Although the activation status of the T cells did not differ, T cells isolated from the lungs and draining lymph nodes of ICOS^+/−^ mice at the peak of inflammation produced less Th2 cytokines upon stimulation *ex vivo*. Using 4get mice, which express GFP upon IL-4 transcription, we determined that the decreased Th2 cytokines in ICOS^+/−^ is due to reduced percentage of Th2 cells and not a defect in their ability to produce IL-4.

**Conclusion:**

These data suggest that in both mice and humans, the level of ICOS surface expression regulates the magnitude of the *in vivo* Th2 response, perhaps by influencing Th2 differentiation.

## Introduction

The steady increase in the number of asthmatic and allergic individuals that react to common allergens in industrialized countries is a major health concern [1]. These atopic individuals have increased allergen specific IgE, total serum IgE and augmented levels of blood eosinophils that are induced by the Th2 subset of T cells. There is a genetic link to the development of atopy and asthma as the concordance rate is significantly higher for monozygotic twins than dizygotic twins [2]. Multiple regions of the genome have been linked with increased atopy in humans including a region on chromosome 2q33. Specifically, this region has been associated with increased eosinophilia, serum IgE and atopy [3]–[5]. Among the candidate genes located in this genomic region that may be responsible for this phenotype are members of the CD28 family of T cell costimulatory molecules including Inducible Costimulator, ICOS.

ICOS is expressed on the surface of T cells and has been implicated in several immune-mediated diseases [6]–[9]. Although ICOS is expressed at low levels on naïve T cells, its surface expression greatly increases upon activation [10], [11] and studies using ICOS-deficient animals have shown that ICOS is critical for Th2 differentiation, germinal center formation and Th2-mediated antibody class switching [12]–[14]. In addition to these ICOS-knockout studies, we and others have shown that blocking ICOS inhibits Th2-mediated lung inflammation, and ICOS blockade *in vitro* resulted in a corresponding decrease in IL-4 and IL-5 production by Th2 cells [15]–[17].

Murine Th2 cells have increased ICOS cell-surface expression compared to Th1 cells [11] and ICOS surface expression levels have been associated with distinct T cell cytokines. For example, T cells expressing ICOS at low levels produced IFN-γ, T cells that expressed ICOS at mid levels produced IL-4 and IL-5, while T cells expressing ICOS at high levels were shown to produce IL-10 [18]. ICOS signaling has also been shown to influence Th2 differentiation by enhancing IL-4 production and IL-4 receptor signaling [10], [19]. Thus, ICOS has a critical role for Th2 differentiation and cytokine production.

We have recently shown that a single nucleotide polymorphism (SNP) in the 5′ human ICOS promoter region associates with increased ICOS surface expression and Th2 cytokine production. Importantly, the same SNP was associated with increased atopy in the Hutterite population [20] suggesting a potential link between ICOS surface expression and Th2 responses. Furthermore, in addition to our findings, both rheumatoid arthritis and lupus patients have higher ICOS cell-surface expression than healthy individuals [21], [22]. However, these models cannot determine if increased ICOS surface expression is contributing to disease progression or alternatively, if it is a result of augmented T cell activation.

To definitively demonstrate whether differences in ICOS cell-surface expression alone can have global effects on a Th2 immune response, we have used a mouse model in which the experimental group had its ICOS surface expression fixed at a lower level than wild-type T cells. We find that ICOS^+/−^ T cells have decreased ICOS surface expression compared to ICOS^+/+^ T cells although activation status was equal, and therefore we used ICOS^+/+^ and ICOS^+/−^ mice to test our hypothesis that genetically decreasing ICOS surface expression levels could directly result in diminished Th2 response. Herein we demonstrate that ICOS^+/−^ mice have decreased Th2 immune responses *in vivo* resulting in decreased airway eosinophilia and defective T cell cytokine production. These results suggest that a genetic predisposition resulting in reduced ICOS expression on the surface of T cells can directly result in diminished Th2 responses.

## Materials and Methods

### Mice

ICOS^−/−^ mice were generated as previously described [12] and backcrossed 8 generations on the C57BL/6 background. ICOS^+/−^ mice were intercrossed to create ICOS^+/+^, ICOS^+/−^ and ICOS^−/−^ littermates. B7RP-1^−/−^ mice were a gift in kind by Andrew Welcher at Amgen. C.129-*Il4^tm1Lky^*/J (4get) mice were purchased from The Jackson Laboratory (Bar Harbor, ME) and bred to BALB/c ICOS^+/−^ mice in our facility. The resulting ICOS^+/+^.4get and ICOS^+/−^.4get littermates were used for the experiments in this manuscript. Animals were housed in a specific pathogen-free facility maintained by the University of Chicago Animal Resources Center (Chicago, IL). The studies detailed herein conform to the principles set forth by the Animal Welfare Act and the National Institutes of Health guidelines for the care and use of animals in biomedical research.

### In vitro stimulation of enriched T cells and flow cytometry

Mice were sacrificed and their lymph nodes were collected and a single cell suspension was made. The cells were incubated with an anti-HSA antibody (J11D, American Type Culture Collection [ATCC], Manassas, VA) for 1 hr at 4°C followed by incubation with sterile-filtered rabbit complement (Pel-Freez, Rogers, AR) for 30 min. at 37°C. Dead cells were removed via centrifugation through Ficoll-Histopaque 1059 (Sigma, St. Louis, MO). The live cells were then stimulated in the presence of plate-bound anti-CD3 and anti-CD28 antibodies for 48 hr in 24 well plates in media alone, IL-4 or anti-IL-4 (11B11). Cells were suspended in 100 µL FACS Buffer (PBS containing 0.1% sodium azide and 1% BSA, and incubated with the anti Fc receptor antibody, 2.4G2 (ATCC), for five minutes at room temperature. Cells were stained with anti-CD3, CD4, ICOS, CD25, CD40L, CD28, CD44 or CD62L antibodies. Flow cytometric analysis was performed on a BD LSRII or FacsCanto (BD Pharmingen, San Diego, CA) and the data analyzed with FlowJo software (Tree Star, Ashland, OR).

### *In vivo* model of airway inflammation

Mice were sensitized and challenged with *S. mansoni* eggs as previously described [16]. Briefly, mice were injected with *S. mansoni* eggs i.p. on day 0, challenged with soluble egg antigen (SEA) intratracheally (i.t.) on day 7, and the mice were sacrificed on day 11. Post sacrifice, blood was isolated via cardiac puncture. Bronchoalveolar lavage (BAL) was performed by delivering 0.8 mL PBS in the airway via a cannulated trachea. The lavage was repeated three times for a final volume of 2.5–3.0 mL and cells were counted using a hemacytometer. The percentage of CCR3^+^ eosinophils, CD4^+^ T cells and CD8^+^ T cells in the BAL were determined using flow cytometry.

In experiments using 4get mice, the mice were injected with *S. mansoni* eggs i.p. on day 0, challenged with soluble egg antigen (SEA) intratracheally (i.t.) on day 7 and day 14, and the mice were sacrificed on day 18. The percentage of GFP^+^ cells were determined by flow cytometry.

### Isolation of lung cells

Lungs were digested by agitating the tissue for 1 hr in 20 mL digestion buffer (collagenase P at 1.0 mg/mL (Boehringer Manheim, Manheim, Germany), DNaseI at 50 U/mL (Boehringer Manheim), hyaluronidase at 85 U/mL (Sigma)). The digest was passed through a nytex filter, and RBCs were depleted with ammonium chloride-potassium lysing buffer. Dead cells were removed via centrifugation through Ficoll-Histopaque 1059 (Sigma).

### Cytokine Analysis

Cells from the lungs and draining lymph nodes were analyzed via ELISPOT or ELISA as described in the results. For ELISPOT, cells were placed on plates coated with either anti-IL-4 or anti-IL-5 antibodies and stimulated overnight in the presence or absence of anti-CD3 antibody. After 24 hours the number of antigen specific spots was analyzed according to the manufacture's instructions (BD Pharmingen). For ELISA, the draining mediastinal lymph nodes were removed, and the cells were stimulated with SEA (5 µg/mL) in 96 well plates at 4×10^5^ cells/mL for 48 hours. Cytokine levels were analyzed via ELISA according to the manufacturer's instructions (IL-5, IFN-γ (BD Pharmingen) and IL-13 (R&D Systems Minneapolis, MN)). In experiments using 4Get mice, cells isolated from both the lungs and mediastinal lymph nodes were stained with anti-CD4 antibodies. The CD4^+^GFP^−^ and CD4^+^GFP^+^ cells were sorted using a FACSAria (BD Pharmingen, San Diego, CA). The number of each cell type was equalized and stimulated in the presence of anti-CD3 and anti-CD28 antibodies for 48 hours and IL-4 production was analyzed via ELISPOT according to the manufacturer's instructions.

### IgE analysis

Blood was isolated from mice and sera was isolated by centrifugation in serum separator tube. IgE levels were analyzed via ELISA according to manufacturer's instructions (BD Pharmingen).

### Statistical Analysis

All statistical analysis was performed using Graphpad Prism software and significance was determined using unpaired student's t test. In all figures and tables, * = *p*<0.05, ** = *p*<0.01, *** = *p<*0.001.

## Results

### ICOS^+/−^ T cells have less cell surface ICOS expression than wild-type T cells

**Figure 1:**
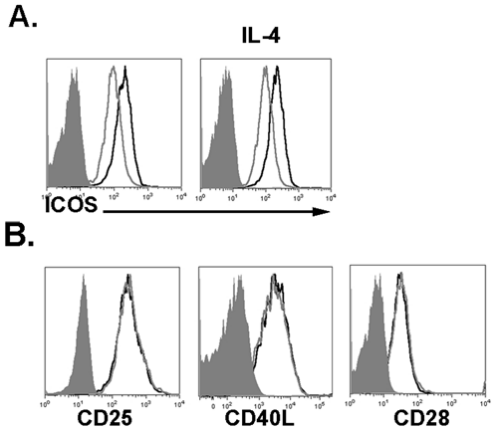
ICOS^+/−^ T cells have decreased ICOS expression but equal activation status. ICOS^+/+^ (black lines) and ICOS^+/−^ (grey line) T cells were isolated and stimulated with anti-CD3 and anti-CD28 antibodies in media alone or in the presence of IL-4 or anti-IL-4 antibodies. After 48 hours the cells were removed and expression of the indicated surface markers were analyzed via flow cytometry. (A) ICOS expression on CD4^+^ T cells stimulated under the indicated conditions. (B) Expression of the indicated cell-surface markers after stimulation for 48 hours in media alone cultures.

To determine if T cells from ICOS heterozygous animals have decreased ICOS expression on their cell surface, T cells were enriched from the lymph nodes from ICOS^+/+^ and ICOS^+/−^ animals and stimulated with plate-bound anti-CD3 and anti-CD28 antibodies. After a 48 hour stimulation, ICOS^+/−^ T cells have decreased ICOS cell-surface expression compared to their wild-type littermates (Fig. 1A). Previous reports have shown that IL-4 may augment ICOS surface expression [23], however, stimulating the cells in the presence of exogenous IL-4 did not significantly alter ICOS surface expression on either ICOS^+/+^ or ICOS^+/−^ T cells (Fig. 1A). Thus, the difference in ICOS surface expression in ICOS^+/−^ T cells was not influenced by IL-4 levels.

Another reason for the decreased ICOS expression on the surface of ICOS^+/−^ could be a result of insufficient activation of the ICOS^+/−^ T cells. However, ICOS^+/−^ T cells did not have decreased surface expression of the activation markers CD25 and CD40L. T cells from ICOS^+/+^ and ICOS^+/−^ mice also express equal levels of CD28, suggesting that disruption of the ICOS gene did not negatively influence the expression of genes near the *ICOS* locus (Fig. 1B). These data indicate that ICOS^+/−^ T cells have decreased capacity to express ICOS compared to ICOS^+/+^ T cells, and this diminished surface expression is not due to a difference in T cell activation or IL-4 production.

### ICOS expression regulates the magnitude of the Th2 immune response

**Figure 2:**
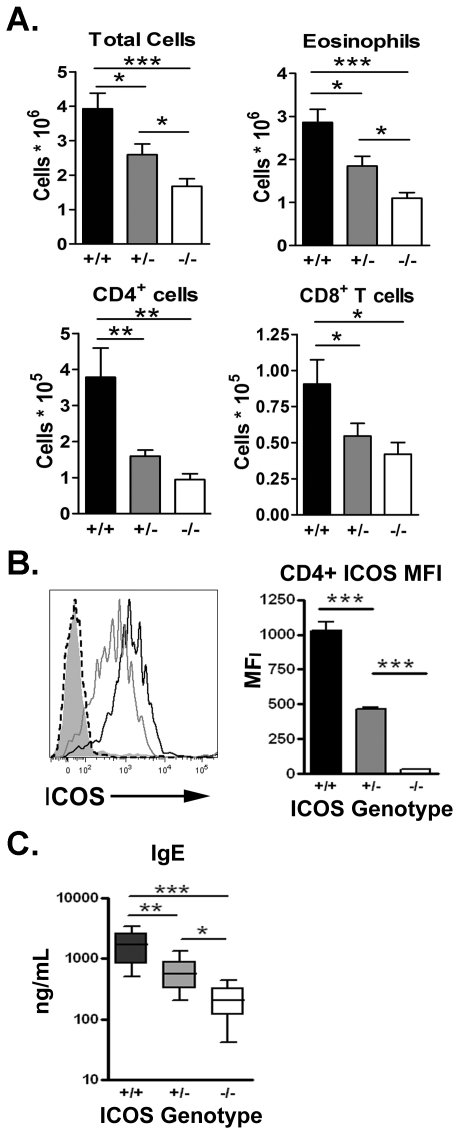
ICOS cell-surface expression regulates the magnitude of a Th2 response *in vivo*. ICOS^+/+^ (black bars), ICOS^+/−^ (grey bars) and ICOS^−/−^ (white bars) mice were activated as indicated in Materials and Methods. (A) Total cells in BAL fluid were counted and percent CCR3^+^ eosinophils, CD4^+^ T cells and CD8^+^ T cells were analyzed via flow cytometry and used to calculate total cell numbers. (B) ICOS expression on BAL CD4^+^ T cells from ICOS^+/+^ (black line), ICOS^+/−^ (grey line), ICOS^−/−^ (dotted line) is shown. (C) Serum IgE levels were analyzed via ELISA.

As ICOS^+/−^ T cells have decreased ICOS expression on their surface upon activation and ICOS has an important role in Th2 responses, we hypothesized that ICOS^+/−^ mice would have decreased Th2-mediated airway inflammation in our model. To test our hypothesis we analyzed the Th2 *in vivo* response of ICOS^+/+^, ICOS^+/−^, and ICOS^−/−^ mice using an established Th2-mediated airway inflammation model [16]. In our model we sensitize the mice i.p. with inactivated *S. mansonii* eggs and challenge the mice with soluble egg antigen (SEA) intratracheally (i.t) resulting in a robust influx of eosinophils into the lungs. We find that ICOS^+/−^ mice had a significant decrease in total cells, eosinophils, CD4^+^ T cells and CD8^+^ T cells in the airways compared to ICOS^+/+^ mice, but ICOS^+/−^ mice still had significantly more total cells and eosinophils as compared to ICOS^−/−^ mice (Fig. 2A). While ICOS^+/−^ mice had a significant reduction of CD4^+^ and CD8^+^ T cells compared to ICOS^+/+^ mice, the CD4^+^ and CD8^+^ T cell response was not significantly different than the ICOS^−/−^ mice. In concordance with our in vitro data, CD4^+^ T cells in the bronchoalveolar lavage (BAL) fluid of sensitized and challenged ICOS^+/−^ mice had a 50% reduction in surface ICOS expression compared to CD4^+^ T cells from ICOS^+/+^ mice (Fig. 2B). Interestingly, we observed a slight but not significant decrease in PAS-positive cells in the airways of ICOS^+/−^ mice compared to ICOS^+/+^ mice suggesting that decreased ICOS expression induces a defect in the cellular immune response (data not shown).

ICOS^−/−^ mice have been shown to have a severe defect in germinal center formation and serum IgE levels [24]. To determine whether decreased ICOS surface expression also affects humoral immune responses, serum IgE levels were analyzed in ICOS^+/−^ mice after sensitization and challenge. We find that ICOS^+/−^ mice have significantly less serum IgE than ICOS^+/+^ mice but still produce significantly more IgE than ICOS^−/−^ mice (Fig. 2C). The difference between ICOS^+/+^ and ICOS^+/−^ mice was not strain specific as BALB/c ICOS^+/−^ mice also had decreased in vivo airway inflammation as compared to BALB/c ICOS^+/+^ mice (data not shown). Together, these data confirm that the level of ICOS costimulation on T cells correlates directly to the severity of Th2 mediated airway inflammation and the levels of serum IgE produced during a Th2 immune response.

### Reduced Th2 response in ICOS^+/−^ mice

**Figure 3:**
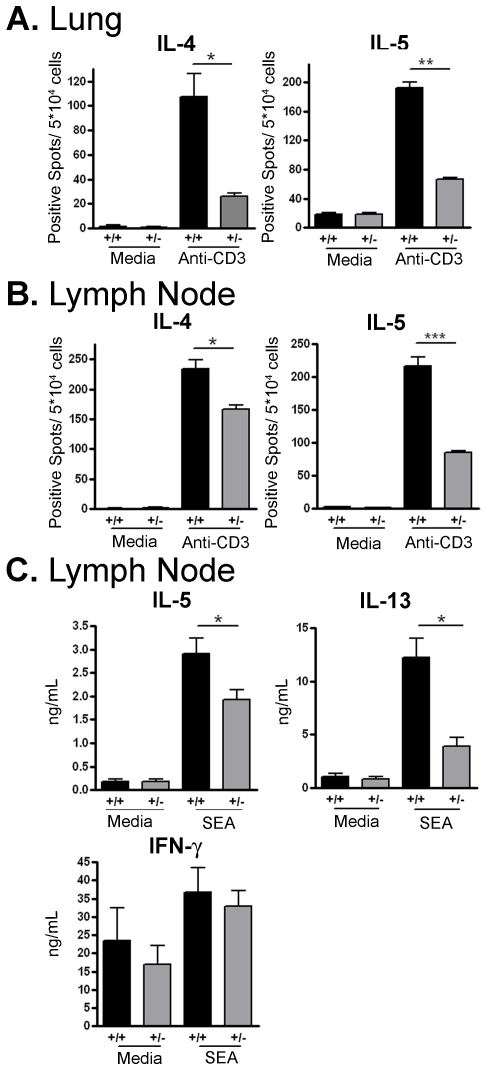
Defective Th2 cytokine production in ICOS^+/−^ mice. (A) Four days after challenge the lungs were harvested and isolated cells were stimulated with anti-CD3 for 24 hours. Number of cells producing IL-4 and IL-5 were quantified via ELISPOT. (B) Mediastinal lymph nodes were stimulated with anti-CD3 for 24 hours, and the number of cells producing IL-4 and IL-5 were quantified via ELISPOT or (C) The cells were stimulated with SEA for 48 hours and cytokine supernatant levels were analyzed via ELISA.

The finding that ICOS^+/−^ mice have decreased lung eosinophilia led us to hypothesize that T cells from these mice have defects in Th2 differentiation and/or effector function. To test this hypothesis, cells from the lung tissue and mediastinal lymph nodes were isolated, and the number of cytokine producing cells was analyzed by ELISPOT after 24 hour stimulation in the presence or absence of anti-CD3 antibodies. ICOS^+/−^ mice had significantly less IL-4 and IL-5 producing cells in both the lungs and draining lymph nodes than ICOS^+/+^ mice (Fig. 3A, B). To determine the cytokine production from antigen-specific T cells, mediastinal lymph nodes cells were stimulated with SEA for 48 hours and their cytokine production was analyzed via ELISA. ICOS^+/−^ T cells had decreased Th2 cytokine production after SEA restimulation but equal IFN-γ production compared to ICOS^+/+^ T cells (Fig. 3C). Although we observed decreased IL-5 and IL-13 production by ICOS^+/−^ T cells, there was not a significant decrease in IL-4 production (ICOS^+/+^ = 200.0±47.1 ng/mL; ICOS^+/−^ = 121.0±33.5 ng/mL). These data suggest a decrease in ICOS expression on ICOS^+/−^ T cells resulted in a proportional reduction in Th2 differentiation and airway inflammation.

The decreased immune response and lung eosinophilia observed in the ICOS^+/−^ mice may also be caused by decreased activation of ICOS^+/−^ T cells. To ensure that the differences observed in Figure 3 were not due to a decreased presence of activated T cells in the ICOS^+/−^ mice, lymphocytes were isolated at the peak of the immune response and analyzed via flow cytometry. ICOS^+/−^ and ICOS^+/+^ mice had similar percentage of CD4^+^ T cells in both the lungs and mediastinal lymph nodes (Table 1). Furthermore, among the CD4^+^ T cells in the lungs and lymph nodes, ICOS^+/+^ and ICOS^+/−^ T cells had an equal percentage of CD44 high cells and CD25^+^ cells. Interestingly, among the CD4^+^ CD44 high cells ICOS^+/−^ T cells have decreased ICOS surface expression compared to ICOS^+/+^ T cells (Table 1). Together, these data indicate that varying the level of surface ICOS expression does not affect global T cell activation *in vivo*.

**Table 1:** Activation and ICOS expression on T cells in ICOS^+/+^ and ICOS^+/−^ mice.

	ICOS^+/+^	ICOS^+/−^
Lymph Node		
Total Cells (×10^6^)	13.6±3.8	10.8±3.6
%CD3^+^	31.7±11.2	32.8±9.6
%CD4^+(a)^	53.9±4.9	49.8±4.3
% CD44^high(b)^	46.5±2.5	41.5±6.8
ICOS MFI(c)	2679±374^***^	1046±123
%CD25^+(b)^	13.8±1.8	12.9±3.6
Lungs		
%CD3^+^	28.0±2.9	26.1±5.5
%CD4^+(a)^	57.2±3.0	53.9±4.0
% CD44^high(b)^	88.8±2.0	82.4±4.8
ICOS MFI(c)	2421.7±242.2^***^	1058.3±101.6

The cell percentages are shown as mean ±− SD as determined via flow cytometry.

(a) percent of CD4^+^ cells within CD3^+^ population.

(b) percent CD44^high^ are within CD3^+^/CD4^+^ population.

(c) ICOS MFI is ICOS expression on CD3^+^/CD4^+^/CD44^high^ cells.

### ICOS downregulates regulates B7RP-1 expression on antigen-presenting cells

**Figure 4:**
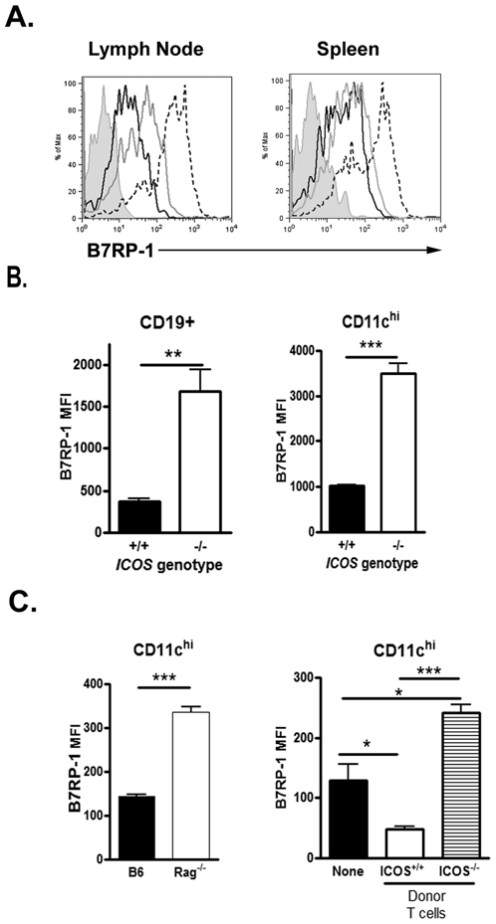
B7RP-1 downregulation on APCs is regulated by ICOS expression. (A) Cells were isolated from the mediastinal lymph node and spleen of ICOS^+/+^ (Black line), ICOS^+/−^ (Grey line), and ICOS^−/−^ (dashed line) mice at the peak of inflammation. The B7RP-1 expression on CD11c^hi^ cells is shown. (B) Cells were isolated from the spleens of naïve mice (ICOS^+/+^ n = 3; ICOS^−/−^ n = 4). The B7RP-1 MFI on the indicated cell types is shown. (C) Left- Cells were isolated from the spleens of naïve C57BL/6 (n = 3) and Rag^−/−^ (n = 3). The B7RP-1 MFI on CD11c^hi^ cells is shown. Right. T cells were isolated from naïve ICOS^+/+^ and ICOS^−/−^ mice and transferred to Rag^−/−^ mice. Eight wks later the mice were sacrificed and B7RP-1 expression was analyzed via flow cytometry.

It is known that ICOS^−/−^ mice have defective Th2 responses, and it has been published that IL-4 can induce B7 Related Protein-1 (B7RP-1) surface downregulation on B cells and peritoneal macrophages [25], [26]. Thus, we hypothesized that antigen presenting cells of ICOS^−/−^ mice would have augmented B7RP-1 expression compared to B6 mice. To test this hypothesis we performed our *S. mansonii*-based airway inflammation model in B6, ICOS^+/−^ and ICOS^−/−^ mice and analyzed the dendritic cell populations of both the mediastinal lymph nodes and spleen at the peak of inflammation. ICOS^−/−^ mice had a dramatic increase in B7RP-1 expression on dendritic cells compared to B6 and ICOS^+/−^ mice (Fig. 4A). ICOS^+/−^ mice had intermediate B7RP-1 expression levels between that of ICOS^+/+^ and ICOS^−/−^ mice suggesting that B7RP-1 expression inversely correlates with ICOS expression. Furthermore, B cells and CD11c^hi^ DC from uninflamed mice also had increased B7RP-1 expression on both cell types in the ICOS^−/−^ mice compared to wild-type mice (Fig. 4B). Hence, in both naïve and inflamed mice ICOS^−/−^ APCs had greatly increased B7RP-1 expression compared to ICOS^+/+^ APCs. However, the B7RP-1 expression on ICOS^+/−^ APCs was only slightly elevated compared to ICOS^+/+^ APCs.

Since the absence of ICOS on T cells results in increased B7RP-1 expression, we tested whether T cells are necessary to reduce B7RP-1 expression by analyzing the B7RP-1 expression in Rag^−/−^ mice. Rag^−/−^ mice lack T cells and therefore lack ICOS^+^ cells. As shown in Fig. 4C, dendritic cells from naïve Rag^−/−^ mice have increased B7RP-1 expression compared to B6 mice further supporting that ICOS expression results in decreased B7RP-1 surface expression. To determine whether it was the absence of T cells or the absence of ICOS expression on T cells that lead to increased B7RP-1 expression, naïve B6 or ICOS^−/−^ T cells were transferred into a naïve Rag^−/−^ mouse and four weeks later, we analyzed the B7RP-1 expression on their dendritic cells. Rag^−/−^ mice which received ICOS-positive B6 T cells had decreased B7RP-1 expression on their splenic dendritic cells as compared to Rag^−/−^ mice which did not receive any cells (Fig. 4D). Interestingly, Rag^−/−^ mice that received ICOS^−/−^ T cells had augmented B7RP-1 expression compared to Rag^−/−^ mice which did not receive any T cells. Thus the transfer of ICOS^−/−^ T cells results in an environment that induces B7RP-1 upregulation. Overall, these data indicate that ICOS surface expression on T cells can regulate B7RP-1 expression on dendritic cells in an active manner.

### Reduced B7RP-1 expression results in diminished Th2 responses

**Figure 5:**
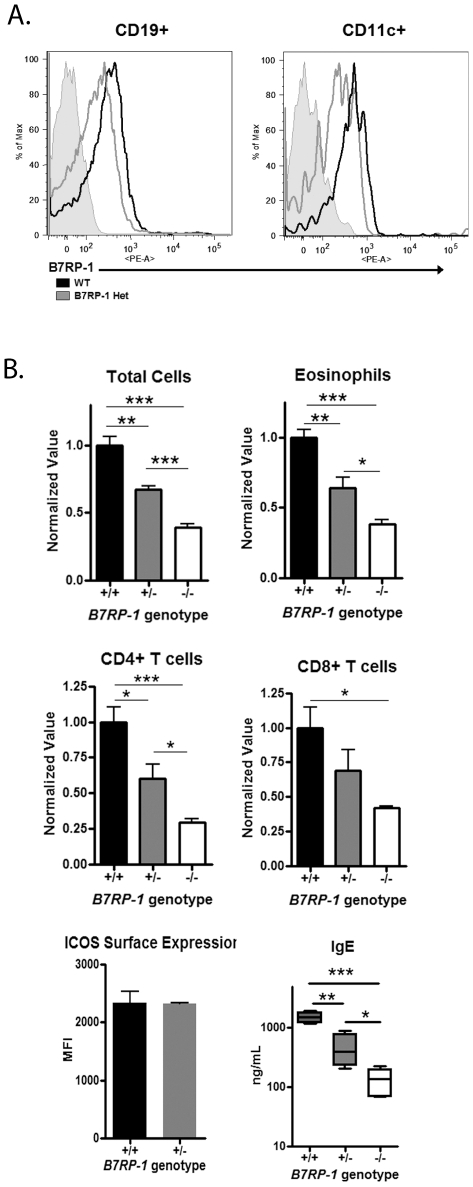
Decreased Th2 response in B7RP-1^+/−^ mice. Splenocytes from Wild-type and B7RP-1^+/−^ mice were removed and single-cell suspensions were made. The cells were stained with B7RP-1 and either CD19 or CD11c. Black = WT Grey = B7RP-1^+/−^. (B) B6. B7RP-1^+/+^ (black bars), B6. B7RP-1^+/−^ (grey bars), and B6. B7RP-1^−/−^ (white bars) mice were sensitized and challenged as indicated in Materials and Methods. Total cells in BAL fluid were counted and percent CCR3^+^ eosinophils, CD4^+^ T cells and CD8^+^ T cells were analyzed via flow cytometry and used to calculate total cell numbers and normalized to the average of the wild-type value. ICOS expression on BAL CD4^+^ T cells isolated from the mediastinal lymph nodes of mice is shown. Serum IgE levels were analyzed via ELISA.

Since ICOS has a single known ligand, B7RP-1, we hypothesized that reduced B7RP-1 expression would result in reduced ICOS costimulation; leading to reduced Th2 responses. We tested this hypothesis by comparing the lung inflammatory response of B7RP-1^+/+^, B7RP-1^+/−^ and B7RP-1^−/−^ mice. To first determine whether B7RP-1^+/−^ mice actually have decreased surface expression on their cells, we analyzed B7RP-1 expression on splenic B cells and dendritic cells. Our results indicate that both CD19^+^ B cells and CD11c^+^ cells from B7RP-1^+/−^ spleens have decreased surface B7RP-1 expression (Fig. 5A) compared to B7RP-1^+/+^ cells.

Since we observe reduced B7RP-1 expression on B cells and monocytes in B7RP-1^+/−^ mice, we performed our airway inflammation model in B7RP-1^+/+^, B7RP-1^+/−^ and B7RP-1^−/−^ mice. Similar to ICOS^+/−^ mice, B7RP-1^+/−^ mice had decreased total cells and eosinophils in the BAL than wild-type mice and significantly increased number of total cells and eosinophils than B7RP-1^−/−^ mice (Fig. 5B). B7RP-1^+/−^ mice also had significantly less CD4^+^ T cells in the BAL compared to wild-type mice. However, B7RP-1^+/−^ mice had significantly more CD4^+^ T cells than B7RP-1^−/−^ mice; different than the ICOS^+/−^ mice which had similar numbers of CD4^+^ T cells compared to ICOS^−/−^ T cells. In concordance to what we observed in ICOS^+/−^ mice, B7RP-1^+/−^ mice also had intermediate serum IgE levels compared to B7RP-1^+/+^ and B7RP-1^−/−^ mice (Fig. 5B). Previous reports and our data suggest B7RP-1 expression is inversely proportional to ICOS expression [27], [28]. Thus, we tested whether B7RP-1^+/−^ mice have altered ICOS expression. CD4^+^ T cells were isolated from the mediastinal lymph nodes of B7RP-1^+/+^ and B7RP-1^+/−^ mice at the peak of inflammation and the level of ICOS expression was analyzed via flow cytometry. The B7RP-1^+/+^ and B7RP-1^+/−^ T cells had similar ICOS surface expression levels; indicating that unlike ICOS expression, reduced B7RP-1 expression does not alter ICOS expression on T cells (Fig. 5B). These data indicate that similar to decreased ICOS expression on T cells, decreased B7RP-1 expression on APCs can also result in diminished Th2-mediated responses in vivo.

### Decreased Th2 immune response in ICOS^+/−^ mice is a result of reduced number of Th2 cells

Our previous experiments determined that ICOS^+/−^ mice have a decreased Th2 response. However, from these experiments we cannot determine whether the difference is due to a reduced number of Th2 cells in the ICOS^+/−^ mice, or if ICOS^+/−^ Th2 cells secrete reduced Th2 cytokines during the effector response. We used the IL-4 Green Enhanced Transcription (4get) mice to determine the difference between these options. We performed an airway inflammation protocol similar to that used in Figure 2 in mice that were ICOS^+/+^.4get and ICOS^+/−^.4get littermates. If reduced ICOS cell-surface expression results in reduced number of Th2 cells, a reduced percentage of CD4^+^GFP^+^ cells should be observed. On the other hand, if decreased ICOS expression results in diminished Th2 cytokine production, the ICOS^+/−^ GFP^+^ T cells will produce less IL-4.

**Figure 6:**
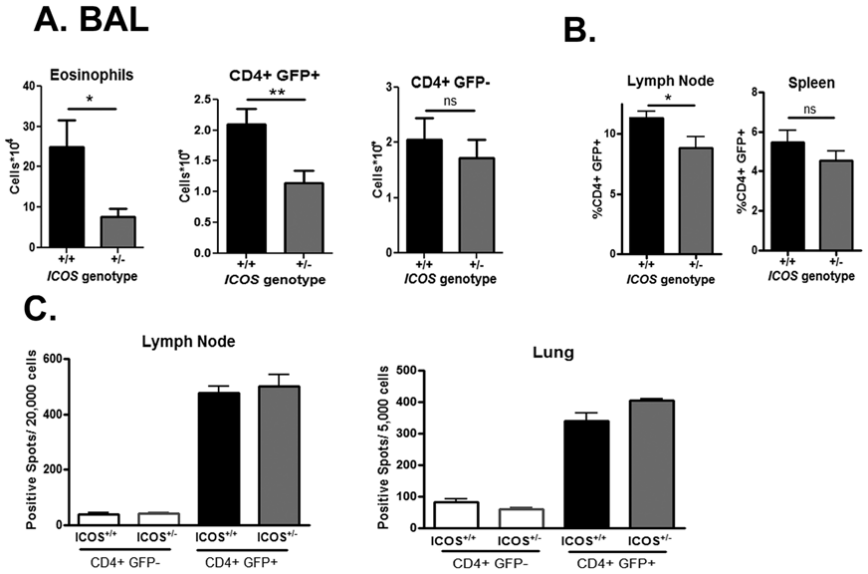
Reduced number of Th2 cells in the airways of ICOS^+/−^ mice. (A) ICOS^+/+^.4get (black bars), ICOS^+/−^.4get (grey bars) mice were activated as indicated in Materials and Methods. Total cells in BAL fluid were counted and percent CCR3^+^ eosinophils, total CD4^+^ T cells, CD4^+^GFP^+^ and CD4^+^GFP^−^ T cells were analyzed via flow cytometry and used to calculate total cell numbers. (B) The percent of CD4^+^GFP^+^ cells from the indicated organs are shown. The results are from separate experiments that have been combined (ICOS^+/+^ n = 7, ICOS^+/−^ n = 9). (C) CD4^+^GFP^−^ and CD4^+^GFP^+^ cells were sorted from the mediastinal lymph nodes and lungs of ICOS^+/+^.4get and ICOS^+/−^.4get mice. The cells were equalized and stimulated with anti-CD3 and anti-CD28 antibodies for 48 hours and the IL-4 production was analyzed via ELISPOT.

The results in Figure 6A indicate that the ICOS^+/−^.4get mice have a significant reduction in the number of eosinophils and CD4^+^GFP^+^ cells in the BAL fluid compared to ICOS^+/+^.4get mice. These results indicate that ICOS^+/−^ mice have fewer cells that have transcribed IL-4 in the airways compared to ICOS^+/+^ mice. Importantly, there are similar numbers of CD4^+^GFP^−^ cells in the BAL, indicating that ICOS^+/−^ mice have a specific defect in the number of eosinophils and Th2 cells in the airways.

In previous results, we demonstrated ICOS^+/−^ mice had decreased Th2 cytokine production in cells isolated from the mediastinal lymph nodes and stimulated ex vivo with the SEA antigen that was used previously to challenge the mice (Fig. 3). Thus, we also analyzed the presence of CD4^+^GFP^+^ cells in ICOS^+/+^.4get mice and ICOS^+/−^.4get mice at the peak of the inflammatory response. Similar to the results observed in the airways, ICOS^+/−^ mice had a significantly reduced percentage of CD4^+^GFP^+^ T cells (Fig. 6B). However, these mice did not have reduced numbers of CD4^+^CD44^high^CD62L^low^ effector cells (20.75%±1.1 vs. 21.22%±2.4). Indicating the difference in Th2 cells in ICOS^+/−^ mice is not due to decreased T cell activation. There was no significant difference in the number of GFP^+^ cells in the spleens of ICOS^+/+^ and ICOS^+/−^ mice (Fig. 6B) indicating that the difference observed was localized to the site of the immune response.

An alternate explanation for the decreased Th2 cytokine production upon ex vivo restimulation is that ICOS^+/−^ T cells have decreased number of IL-4 transcripts on a per cell basis. To determine if ICOS^+/−^ Th2 cells also have decreased IL-4 transcription on a per cell basis, we compared the GFP MFI of the CD4^+^GFP^+^ cells between the two groups. ICOS^+/+^ and ICOS^+/−^ T cells in both the BAL and mediastinal lymph nodes have similar GFP MFI (data not shown).

To confirm the notion that equal ICOS^+/+^ GFP^+^ and ICOS^+/−^ GFP^+^ cells have similar capacity to produce IL-4, we sorted CD4^+^GFP^+^ and CD4^+^GFP^−^ cells from the lungs and mediastinal lymph nodes of ICOS^+/+^ and ICOS^+/−^ 4get mice at the peak of inflammation, equalized their numbers and restimulated them with anti-CD3 and anti-CD28 to analyze their IL-4 production. In contrast to Figure 3 where we stimulated all whole lung and mediastinal lymph nodes, when we normalized the number of cells, ICOS^+/−^ T cells did not have reduced IL-4 production (Figure 6C). Therefore, our data suggests that the decreased lung inflammatory response observed in the ICOS^+/−^ mice is due to reduced number of Th2 cells and not in the ability of those Th2 cells to produce cytokines.

## Discussion

Multiple studies have shown that ICOS is important for Th2 immune responses and cytokine production [12]–[14]. The majority of these studies compare ICOS^+/+^ and ICOS^−/−^ mice, but to date only a small number of ICOS-deficient patients have been identified [29]. Our novel study comparing the immune responses between ICOS^+/+^ and ICOS^+/−^ mice bears broader physiological relevance to human diseases in which variances in ICOS expression levels have been found. Tafuri et al. demonstrated that ICOS^+/−^ mice have decreased serum antibodies [14], but did not explore the influence of T cell ICOS surface expression on tissue inflammation. Our data adds to the study by Tafuri et al. and demonstrates that the decreased ICOS cell-surface expression leads to decreased lung eosinophilia, numbers of Th2 cells, and Th2 cytokine production. To our knowledge, this is the first study demonstrating that reducing the expression of a single costimulatory molecule alone (versus total deletion) can have a significant effect on a tissue-specific Th2 immune response without influencing T cell activation. Yagi et al. showed that ICOS expression varies between numerous murine strains and there is a positive correlation between ICOS expression and IL-4 production across strains [23]. However, there are numerous genetic differences between the strains that may influence both ICOS expression and Th2 differentiation. In this report we have minimized the background genetic differences outside of ICOS surface expression by using littermate ICOS^+/+^ and ICOS^+/−^ mice; thus any defects observed are due to differences in this specific costimulatory pathway and not background genetic differences.

To explore how the level of ICOS-B7RP-1 interactions can regulate Th2 responses, we used a model in which ICOS cell-surface expression levels are pre-determined and compared the responses of ICOS^+/+^ mice and ICOS^+/−^ mice in an established airway inflammation model. We demonstrate that ICOS^+/−^ mice had less surface ICOS expression on CD4^+^ T cells than ICOS^+/+^ T cells. The reduction in ICOS surface expression did not alter global T cell activation but had a substantial influence on Th2 immune responses *in vivo*. The ICOS^+/−^ mice had decreased cellular infiltrates in the airways, decreased airway eosinophilia, decreased serum IgE, and defective Th2 cytokine production upon in vitro restimulation of T cells from the lungs and draining lymph nodes. These results show the reduced ICOS surface expression observed in the ICOS^+/−^ mice leads to defective Th2 immune responses. Since both IL-5 and IL-13 are classical Th2 cytokines and potent activators of eosinophil differentiation and recruitment, the decrease in these cytokines likely contributes to the decrease in airway eosinophilia observed in the ICOS^+/−^ mice. By using this model we are able to demonstrate that reduction in ICOS expression on the surface of T cells resulted in reduced Th2 responses *in vivo*.

ICOS expression also regulates B7RP-1 expression in addition to Th2 cytokine responses. We observed that B6 dendritic cells and B cells have decreased B7RP-1 surface expression compared to ICOS^−/−^ DCs in both untreated and inflamed mice; hence, providing new insight into the ability of T cells to regulate dendritic cells. Interestingly, the B7RP-1 expression on ICOS^+/−^ APCs is slightly augmented but similar to ICOS^+/+^ APCs and is greatly reduced compared to ICOS^−/−^ APCs. Thus we believe the decreased Th2 response observed in ICOS^+/−^ mice is not due to differences in B7RP-1 expression. It is often discussed how naïve T cells need to interact with dendritic cells in the periphery to survive via interactions between the TCR and MHC. However, my data suggests that the T cell/dendritic cell interaction can also influence the dendritic cells and the presence or absence of ICOS can change the status of the resident DCs. Therefore, these data provide unique insights into the communication between T cells and dendritic cells.

We utilized the 4get mice that express GFP upon IL-4 transcription to determine at what stage during the immune response that decreased cell surface ICOS expression by ICOS^+/−^ T cells lead to decreased Th2 responses. The ICOS^+/−^ mice had fewer CD4^+^ GFP^+^ cells in both the airways and mediastinal lymph nodes, but when the number of GFP^+^ CD4^+^ T cells isolated from ICOS^+/+^ and ICOS^+/−^ mice were equalized, the cells produced similar amounts of IL-4. These data indicate that if an ICOS^+/−^ T cell becomes a Th2 cell it can transcribe IL-4 as well as an ICOS^+/+^ Th2 cell. However, there are fewer Th2 cells in the ICOS^+/−^ mice compared to ICOS^+/+^ mice indicating that ICOS cell-surface expression levels regulate Th2 differentiation *in vivo*. Previous reports have demonstrated that 4get T cells with increased GFP expression also have increased IL-4 protein expression as indicated by intracellular staining [30]. Therefore, it is reasonable to presume that the ICOS^+/+^.4get and ICOS^+/−^.4get T cells that are GFP^+^ have similar IL-4 producing capabilities. From our data, we conclude that ICOS^+/−^ mice have a decreased effector response due to decreased number of Th2 cells but not a defect in the cytokine production of effector T cells.

The human genome project demonstrated that the majority of the differences among humans were due to SNPs in the genome, and less than 1% of the SNPs are predicted to result in a mutation in protein function [31]. It is likely that SNPs in the regulatory regions of genes will influence protein expression and this difference in protein expression will have a dramatic impact on the individual. In our model there is a 50% reduction in ICOS surface expression on the T cells of ICOS^+/−^ mice. This difference results in a 50% reduction in the number of Th2 cells in the BAL and a significant reduction in the percentage of Th2 cells in the mediastinal lymph nodes. This difference in Th2 cells may have greater consequences. For example, previous reports have indicated that IL-4 can upregulate eotaxin expression [32], [33], which will recruit eosinophils into the lung. Thus, a reduction in the number of Th2 cells in the lungs leads to a decreased number of eosinophils that arrive in the lung. These eosinophils can augment the existing Th2 response by secreting their own IL-4. Similar to other reports, we observed that the eosinophils in the BAL in the 4get mice are greater than 95% GFP^+^ (data not shown, [34]). Remarkably, this large difference in eosinophils and Th2 cytokine production all results from a small difference in ICOS surface expression.

Interestingly, the defect in the number of Th2 cells in ICOS^+/−^ was only observed at the site of the immune response as there were equal numbers of CD4^+^ GFP^+^ cells in the spleens of ICOS^+/+^ and ICOS^+/−^ mice. We are currently investigating whether, in addition to their defect in Th2 differentiation, ICOS^+/−^ mice also have a defect in Th2 migration that may also contribute to the reduced number of CD4+ GFP+ cells observed in the airways of ICOS^+/−^ mice. It has previously been demonstrated that IL-4 can contribute to the induction of the Th2 chemokine, CCL17, in both human and murine lung epithelial cells [35], [36]. Therefore, the defect in Th2 differentiation in ICOS^+/−^ mice may also result in decreased chemotaxis of existing Th2 cells to the site of inflammation.

We recently published that in humans an *ICOS* promoter region single nucleotide polymorphism is associated with allergy and increased serum IgE in a founder population [20]. The SNP also correlates with increased ICOS surface expression and increased Th2 cytokine production from peripheral blood mononuclear cells. The data in this report provides a possible functional explanation for the findings in Shilling et al. and suggests that a genetic predisposition to express higher ICOS levels is a marker for an augmented Th2 inflammatory response, and this augmented response may lead to Th2-mediated diseases such as atopy. A recent report identified a novel ICOS repressor named Roquin [37]. The *sanroque* mice, which lack Roquin resulting in augmented ICOS cell-surface expression, acquire a lupus like phenotype characterized by increased dsDNA antibodies, increased serum IgG and IgE and splenomegaly. Thus, in both mice and humans, the level of ICOS surface expression regulates the magnitude of the *in vivo* Th2 response, perhaps by influencing Th2 differentiation. Furthermore, genetic predisposition to express higher levels of costimulatory molecules may result in differences in likelihood of developing Th2-type diseases.

Since there is a correlation between ICOS surface expression and in vivo responses, ICOS blockade may be a possible therapeutic target to inhibit diseases initiated by immune hyperresponsiveness. A critical component of rheumatoid arthritis, lupus and myasthemia gravis is the production of anti-self antibodies. Because ICOS costimulation is associated with enhanced B cell isotype switching, blockade of the ICOS/B7RP-1 protein may effectively inhibit the production of harmful anti-self antibodies and reduce disease severity. These data indicate that partially blocking ICOS-B7RP-1 interactions may be an effective therapy for this and other chronic diseases. The partial blockade of this pathway during a hyperactive Th2 immune response may reduce Th2 cytokine production without completely debilitating the immune response, allowing the immune system to maintain a normal immune response against pathogens.
